# Particulate Matter Air Pollution Exposure, Distance to Road, and Incident Lung Cancer in the Nurses’ Health Study Cohort

**DOI:** 10.1289/ehp.1307490

**Published:** 2014-06-03

**Authors:** Robin C. Puett, Jaime E. Hart, Jeff D. Yanosky, Donna Spiegelman, Molin Wang, Jared A. Fisher, Biling Hong, Francine Laden

**Affiliations:** 1 Maryland Institute for Applied Environmental Health, University of Maryland School of Public Health, College Park, Maryland, USA; 2 Channing Division of Network Medicine, Brigham and Women’s Hospital and Harvard Medical School, Boston, Massachusetts, USA; 3 Department of Environmental Health, Harvard School of Public Health, Boston, Massachusetts, USA; 4 Department of Public Health Sciences, Pennsylvania State University College of Medicine, Hershey, Pennsylvania, USA; 5 Department of Epidemiology, and; 6 Department of Biostatistics, Harvard School of Public Health, Boston, Massachusetts, USA; 7 Department of Medicine, Harvard Medical School, Boston, Massachusetts, USA; * These authors contributed equally to this manuscript.

## Abstract

Background: A body of literature has suggested an elevated risk of lung cancer associated with particulate matter and traffic-related pollutants.

Objective: We examined the relation of lung cancer incidence with long-term residential exposures to ambient particulate matter and residential distance to roadway, as a proxy for traffic-related exposures.

Methods: For participants in the Nurses’ Health Study, a nationwide prospective cohort of women, we estimated 72-month average exposures to PM_2.5_, PM_2.5–10_, and PM_10_ and residential distance to road. Follow-up for incident cases of lung cancer occurred from 1994 through 2010. Cox proportional hazards models were adjusted for potential confounders. Effect modification by smoking status was examined.

Results: During 1,510,027 person-years, 2,155 incident cases of lung cancer were observed among 103,650 participants. In fully adjusted models, a 10-μg/m^3^ increase in 72-month average PM_10_ [hazard ratio (HR) = 1.04; 95% CI: 0.95, 1.14], PM_2.5_ (HR = 1.06; 95% CI: 0.91, 1.25), or PM_2.5–10_ (HR = 1.05; 95% CI: 0.92, 1.20) was positively associated with lung cancer. When the cohort was restricted to never-smokers and to former smokers who had quit at least 10 years before, the associations appeared to increase and were strongest for PM_2.5_ (PM_10_: HR = 1.15; 95% CI: 1.00, 1.32; PM_2.5_: HR = 1.37; 95% CI: 1.06, 1.77; PM_2.5–10_: HR = 1.11; 95% CI: 0.90, 1.37). Results were most elevated when restricted to the most prevalent subtype, adenocarcinomas. Risks with roadway proximity were less consistent.

Conclusions: Our findings support those from other studies indicating increased risk of incident lung cancer associated with ambient PM exposures, especially among never- and long-term former smokers.

Citation: Puett RC, Hart JE, Yanosky JD, Spiegelman D, Wang M, Fisher JA, Hong B, Laden F. 2014. Particulate matter air pollution exposure, distance to road, and incident lung cancer in the Nurses’ Health Study Cohort. Environ Health Perspect 122:926–932; http://dx.doi.org/10.1289/ehp.1307490

## Introduction

A number of general population studies around the world have demonstrated adverse associations of chronic exposures to ambient particulate matter (PM) and/or traffic-related pollutants with lung cancer (Beelen et al. 2008a, 2008b; Beeson et al. 1998; Cao and Gao 2012; Carey et al. 2013; Cesaroni et al. 2013; Hales et al. 2013; Hart et al. 2011; Heinrich et al. 2013; Hystad et al. 2013; Jerrett et al. 2013; Katanoda et al. 2011; Krewski et al. 2009; Lepeule et al. 2012; Lipsett et al. 2011; McDonnell et al. 2000; Naess et al. 2007; Nafstad et al. 2003; Nyberg et al. 2000; Pope et al. 2002; Raaschou-Nielsen et al. 2010, 2011, 2013; Turner et al. 2011). Many of these studies have observed effect modification by smoking status, providing evidence for the link between PM exposure and lung cancer in the absence of the strong influence of smoking behavior. Based primarily on the findings of these studies and evidence from occupationally exposed populations, the International Agency for Research on Cancer (IARC) has recently declared outdoor air pollution generally, and PM specifically, as Group 1 human carcinogens (Loomis et al. 2013).

To date, the pollutants/exposures examined and the time periods and spatial scale of those exposures have been somewhat inconsistent across the literature. Most studies in the literature have focused primarily on PM ≤ 10 μm in aerodynamic diameter (PM_10_) or ≤ 2.5 μm (PM_2.5_); however, a number have also considered black carbon/black smoke, nitrogen dioxide (NO_2_), nitrogen oxides (NO_x_), sulfure dioxide, ozone, and volatile organic compounds (Beelen et al. 2008a, 2008b; Dockery et al. 1993; Filleul et al. 2005; Heinrich et al. 2013; Jerrett et al. 2013; Krewski et al. 2009; Nafstad et al. 2003; Nyberg et al. 2000; Raaschou-Nielsen et al. 2010, 2011; Villeneuve et al. 2013; Vineis et al. 2006). A few studies have focused on traffic exposures: modeling NO_2_ from traffic sources alone (Nafstad et al. 2003; Nyberg et al. 2000; Raaschou-Nielsen et al. 2010, 2011) or using distance to major roadways or traffic volume surrounding a location (Beelen et al. 2008a, 2008b; Cesaroni et al. 2013; Hystad et al. 2013; Raaschou-Nielsen et al. 2011, 2013; Vineis et al. 2006). Many studies have relied on area-level assessment of exposure; however, some have also modeled air pollution at the residential level with the intent to decrease measurement error. Studies have also used a variety of periods of exposure relative to disease diagnosis, given that the relevant time period of exposure is unknown. Furthermore, with a few exceptions (Cao and Gao 2012; Lipsett et al. 2011), potential confounders have been assessed only once, even in prospective cohort studies. Despite these inconsistencies in the current body of literature, a link between lung cancer and ambient air pollution has been demonstrated.

The current study is based in the United States within the all-female Nurses’ Health Study (NHS) cohort. Our objective is to examine the association of lung cancer incidence with residential-level chronic exposure to PM_2.5_, PM between 2.5 and 10 μm in diameter (PM_2.5–10_), PM_10_, and residential distance to road. With a wealth of time-varying information on exposures and potential confounders, this cohort provides a unique opportunity to examine these associations.

## Methods

*Study population*. The NHS is an ongoing prospective cohort of 121,700 female nurses who were enrolled in 1976 when they were between 30 and 55 years of age. Participants initially were recruited from 11 states, but as of the mid-1990s nurses now reside in each of the 50 states. A map of all residential addresses in the 48 contiguous states is presented in the Supplemental Material, Figure S1. Participants complete mailed biennial questionnaires to provide information on potential risk factors and to self-report new diagnoses of health outcomes. The response rates are > 90% for each follow-up cycle. Vital status is ascertained through next of kin and the National Death Index (http://www.cdc.gov/nchs/ndi.htm); both methods have identified an estimated 98% of deaths in the cohort. The analytical population for this study excluded all women who were dead or had a previous diagnosis of cancer (except for non-melanoma skin cancer) before follow-up or did not have information for the exposures of interest. The study was approved by the Internal Review Board of Brigham and Women’s Hospital; and informed consent was implied through return of the questionnaires. In addition, this study was approved by the Connecticut Department of Public Health (DPH) Human Investigations Committee. Certain data used in this publication were obtained from the DPH.

*Case ascertainment*. Lung cancers were self-reported by the participants or next of kin or were identified from death certificates; and first reports were subsequently confirmed with medical records by physicians blinded to exposure status. Medical records were obtained for 83% of reported cases; of those, 87% had primary lung cancer confirmed by pathology reports. However, because lung cancers were well reported in this cohort, we included any primary report reconfirmed by the participant where pathological reports were not available.

*Exposure assessment*. As part of the questionnaire mailing process, residential address information is updated every 2 years. All available addresses (1976, 1986–2010) have been geocoded to obtain the corresponding latitude and longitude. For women with a street segment–level geocode (i.e., highest quality, 80–90% of the available addresses in each follow-up cycle), we calculated distance to road at each address as a proxy for traffic-related exposures. Distance to the nearest road (meters) was determined using geographic information system (GIS) software (ArcGIS, version 9.3; ESRI, Redlands, CA) and the ESRI Streetmap Pro2007 data set. We calculated the shortest distances to the following road classes as defined by the U.S. Census Bureau (2001): A1 (primary roads, typically interstate highways, with limited access, division between the opposing directions of traffic, and defined exits), A2 (primary major, non-interstate highways and major roads without access restrictions), and A3 (smaller, secondary roads, usually with more than two lanes). Analyses were conducted using distance to the nearest of all three road types (A1–A3), distance to the two largest road types (A1, A2), and distance to the largest road type (A1). Given the distribution of distance to road in this cohort and previous exposure studies showing approximately exponential decay in exposures to traffic-related air pollutants with increasing distance from a road, we divided distance to road into the following categories: 0–50, 50–200, and ≥ 200 m (Adar and Kaufman 2007; Karner et al. 2010; Lipfert and Wyzga 2008; Lipfert et al. 2006, 2008; Sahlodin et al. 2007; Zhu et al. 2002). We also considered analyses of continuous distance to roads.

Ambient GIS-based spatiotemporal exposure model predictions of PM_2.5_ and PM_10_ were available for all months between January 1988 and December 2007 for the continental United States. These values were generated for each address from nationwide expansions of previously validated spatiotemporal models (Weuve et al. 2012; Yanosky et al. 2008, 2009, in press). The models used monthly average PM_2.5_ and/or PM_10_ data from the U.S. Environmental Protection Agency’s (EPA) Air Quality System (U.S. EPA 2009), the IMPROVE network (Visibility Information Exchange Web System 2009) and various other sources (Spengler et al. 1996; Suh et al. 1997). Generalized additive mixed models with monthly penalized spline smooth spatial terms, penalized spline smooth terms of geospatial predictors (listed below), and terms for time were used to create separate PM prediction surfaces for each month and each PM size fraction (Yanosky et al. 2009). The following geospatial predictors used by the models were generated using a GIS: distance to nearest A1–A4 roads, percent urban land use within 1 km, elevation, point sources of PM (PM_2.5_ emissions density within 7.5 km for PM_2.5_ models, and PM_10_ emissions density within 7.5 km for PM_10_ models), smoothed county population density, tract population density (only for PM_10_), and meteorological predictors: wind speed, total precipitation, temperature, and percent stagnant air days per month (Yanosky et al. 2009). Because monitoring data on PM_2.5_ are limited before 1999, PM_2.5_ in the period before 1999 was modeled using data on PM_10_ (Yanosky et al. 2008). By subtraction of the monthly PM_10_ and PM_2.5_ estimates, information was also obtained on PM_2.5–10_. Cross-validation results demonstrated that the models had high predictive accuracy (cross-validation *R*^2^ values of 0.59, 0.76, and 0.77 for PM_10_, pre-1999 PM_2.5_, and post-1999 PM_2.5_, respectively).

*Potential confounders and effect modifiers*. We selected *a priori* a number of potential confounders or effect modifiers previously associated with lung cancer or exposure in this cohort. Information on the following time-varying variables was available every 2 or 4 years from the follow-up questionnaires: body mass index (BMI; kilograms per meter squared, continuous), physical activity in metabolic equivalent hours per week (MET hr/week; < 3, 3 to < 18, ≥ 18), overall diet quality (Alternative Health Eating Index, continuous) (Chiuve et al. 2012), alcohol consumption (dichotomized at 0 g/day), smoking status (current, former, never), months since quitting for former smokers (continuous), and pack-years (continuous). In 1982 a question was included on exposure to secondhand smoke at home and work and during childhood. We considered census-tract median household income and median house value as measures of area-level socioeconomic status (SES). To account for differences in exposure and other unmeasured regional factors, we also controlled for U.S. geographic region of residence (Northeast, South, Midwest, West).

*Statistical analysis*. Time-varying Cox proportional hazards models were used to assess the relationship of incident lung cancer with residential distance to road and exposure to PM_2.5_, PM_10_, or PM_2.5–10_. Hazard ratios (HRs) and 95% CIs were calculated for each category of roadway proximity compared with the furthest category. We examined the linearity of the association with distance to road using cubic splines, and considered models examining the linear dose response with distances of 0–499 m, compared with values ≥ 500 m. For the metrics of PM we calculated HRs and 95% CIs for a 10-μg/m^3^ increase in each size fraction, after assessing linearity of the dose response. Because the appropriate averaging period is unknown, yet assumed to be more chronic than short-term, we considered 24-, 48-, 72-, 96-, and 120-month cumulative averages. We used Akaike’s information criterion (AIC) (Akaike 1974) to determine the best fit cumulative average for PM_2.5_ among individuals with at least 120 months of exposure, so that the AIC criteria were evaluated among a single population. In sensitivity analyses we considered the consistency of results for other possible averaging times. A *p*-value of 0.05 was used to determine statistical significance.

In the Cox models, person-months were calculated from the start of disease follow-up until June 2010, the end of follow-up, censoring at event, death from another cause, or loss to follow-up, whichever occurred first. We determined the start of disease follow-up based on the selected cumulative average. All models were based on a biennial time scale, were stratified by age in months and time period, and were adjusted for geographic region. Separate models were run for the distance to road types and for each size fraction of PM. Potential confounders (or sets of confounders) were entered separately into the basic model to determine their influence on the association of the exposure with lung cancer. We included variables in the final models that changed the effect estimate for PM_2.5_ and lung cancer at least 10%. For comparability, we used the same confounders across the different size fractions and distance to road models.

To examine effect modification by smoking status, we performed stratified models and created multiplicative interaction terms. Because of small numbers of cases among never-smokers, we also considered effect modification combining never-smokers with former smokers who had quit at least 10 years previously (“long-term former smokers”). Sensitivity analyses were performed restricted to nonmovers, defined as women who remained at the same address between 1976 and the start of follow-up. SAS version 9.2 (SAS Institute Inc., Cary, NC) was used for all analyses.

## Results

Based on the assessment of averaging times described in the methods, the 72-month average was identified as the optimal cumulative average, and therefore we began disease follow-up for all analyses in 1994. A total of 103,650 participants were available for analysis of PM exposures (4,548 died before 1994, 10,710 had a previous diagnosis of cancer, and an additional 2,753 had no information on air pollution).

Age-adjusted characteristics during follow-up are presented overall and by smoking status in Table 1. The mean (± SD) age was 67.0 ± 8.3 years, the age-adjusted mean BMI was 25.6 ± 7.5 and about 39% reported between 3 and 18 MET hr/week of physical activity. About half of the women lived in the Northeast, and 52% of participants reported secondhand smoke exposure at work and from their parents and about 35% at home. More never-smokers were nondrinkers and were less exposed to secondhand smoke, whereas more current smokers reported < 3 MET hr/week of physical activity. Distributions of the three size fractions of PM overall and by region and correlations between the three size fractions within and across cumulative averages are presented in Supplemental Material, Tables S1 and S2, respectively.

**Table 1:** Age-adjusted descriptive characteristics averaged over follow-up (1994–2010) among 103,650 participants in the Nurses’ Health Study overall and ­stratified by smoking status.

Characteristic	All participants	Never-smokers	Former smokers	Current smokers
Person-years (%)^*a*^	1,510,027 (100)	668,581 (44)	663,062 (44)	175,563 (12)
Age [years (mean ± SD)]^*a*^	67.0 ± 8.3	67.1 ± 8.5	67.4 ± 8.2	64.8 ± 7.9
BMI [kg/m^2^ (mean ± SD)]	25.6 ± 7.5	25.8 ± 7.4	26.4 ± 6.8	22.1 ± 9.4
Pack-years of smoking (mean ± SD)	13.4 ± 20.0	0.0 ± 0.0	19.3 ± 18.5	43.0 ± 23.0
Months since quit smoking (mean ± SD)	123.9 ± 178.8	0.0 ± 0.0	279.2 ± 167.4	0.0 ± 0.0
Alternative healthy eating index (mean ± SD)	180.4 ± 108.5	182.8 ± 108.0	190.6 ± 106.5	129.4 ± 102.1
Census-tract median household income (mean ± SD)	63,518 ± 24,491	62,648 ± 23,194	64,849 ± 24,978	61,954 ± 23,644
Census-tract median home value (mean ± SD)	170,126 ± 125,261	166,431 ± 123,720	176,055 ± 128,455	161,998 ± 118,139
Moved between 1976 and 1994 (%)^*a*^
No	65.4	66.1	64.1	67.4
Yes	34.6	33.9	35.9	32.6
Region (%)
Northeast	51.1	48.0	53.2	54.6
Midwest	17.3	19.4	15.4	16.4
West	13.7	14.5	13.3	11.4
South	18.0	18.1	18.1	17.7
Alcohol category (%)
Nondrinker (0 g/day)	15.0	22.1	9.3	9.7
Drinker	71.4	63.3	80.3	68.5
Missing	13.6	14.6	10.4	21.8
Physical activity (%)
< 3 MET hr/week	21.5	20.5	21.2	26.0
3 to < 18 MET hr/week	38.8	39.7	39.4	33.2
≥ 18 MET hr/week	30.7	31.2	33.0	20.3
Missing	9.0	8.5	6.5	20.5
Secondhand smoke during childhood (%)
None	25.1	30.9	21.8	15.7
From mother	3.8	3.1	4.4	3.8
From father	33.9	34.1	34.9	29.1
From both parents	14.7	11.2	17.5	17.0
Missing	22.6	20.7	21.4	34.4
Home secondhand smoke (%)
None	33.2	38.6	31.8	17.7
Occasional	18.6	18.7	19.6	14.0
Regular	17.1	11.4	19.5	29.6
Missing	31.2	31.3	29.1	38.8
Occupational secondhand smoke (%)
None	15.0	16.9	14.5	9.5
Occasional	29.3	33.0	28.6	17.7
Regular	22.6	19.2	24.5	28.1
Missing/not working	33.1	30.9	32.4	44.7
Women can be in multiple smoking status categories throughout follow-up. ^***a***^Not age-adjusted.				

All of our *a priori* potential confounders met our definition of confounding and were included in the final multivariable adjusted models. Physical activity, diet, and census-tract median income and median home value (U.S. Census Bureau 2001) attenuated the effect estimate when added to the basic models, whereas estimates increased after control for alcohol consumption and BMI. We considered a number of combinations of smoking variables, including smoking status and pack-years; smoking status, pack-years, and months since quitting; smoking status, cigarettes per day, and duration of smoking; and smoking status, number of cigarettes per day, duration of smoking, and months since quitting. Adjustment for any of these combinations led to similar elevated effect estimates (data not shown); therefore, we included smoking status, pack-years, and months since quitting in all multivariable models.

There were a total of 1,510,027 person-years of follow-up and 2,155 lung cancer cases (1,930 definite) among the 103,650 women with information on 72-month cumulative average PM. Models of associations between lung cancer and 72-month average exposures to the different size fractions of PM are presented in Table 2. There were no statistically significant deviations from linearity; therefore, we present linear exposure–response functions. In basic models adjusted for age, calendar time, and region of the country, each 10-μg/m^3^ increase in PM_10_, PM_2.5_, and PM_2.5–10_ was associated with positive, but nonstatistically significant, HRs in the full cohort. The HRs from the multivariable models were similar to those from the basic models for PM_10_ and PM_2.5_; however, the HRs in multivariable models for PM_2.5–10_ were attenuated compared with those in the basic models. For all size fractions, the magnitude of the HRs increased in models restricted to never-smokers. There was a suggestion of effect modification comparing never-smokers and former smokers who had quit at least 10 years earlier with current smokers or former smokers who had quit < 10 years earlier (*p* for interaction: for PM_10_, 0.09; for PM_2.5_, 0.09; for PM_2.5–10_, 0.26). For example, for each 10-μg/m^3^ increase in PM_2.5_, among never-smokers and former smokers who had quit at least 10 years earlier, the HR was 1.37 (95% CI: 1.06, 1.77), whereas among current and recent former smokers the HR was 0.94 (95% CI: 0.76, 1.15).

**Table 2:** HRs (95% CIs) of the association of incident lung cancer 1994–2010 per 10‑μg/m^3^ increase in 72-month cumulative average PM exposures among 103,650 members of the Nurses’ Health Study.

Case definition/cohort	Cases	Person-years	PM_10_		PM_2.5_		PM_2.5–10_	
			Basic^*a*^	Adjusted^*b*^	Basic^*a*^	Adjusted^*b*^	Basic^*a*^	Adjusted^*b*^
All cases
Full cohort	2,155	1,510,027	1.06 (0.98, 1.16)	1.04 (0.95, 1.14)	1.05 (0.90, 1.23)	1.06 (0.91, 1.25)	1.12 (0.98, 1.27)	1.05 (0.92, 1.20)
Never-smokers	176	668,581	1.12 (0.85, 1.46)	1.11 (0.85, 1.46)	1.24 (0.74, 2.05)	1.25 (0.75, 2.07)	1.13 (0.75, 1.70)	1.11 (0.74, 1.68)
Never or quit smoking ≥ 10 years	828	1,203,946	1.11 (0.97, 1.28)	1.15 (1.00, 1.32)	1.22 (0.95, 1.57)	1.37 (1.06, 1.77)	1.11 (0.91, 1.37)	1.11 (0.90, 1.37)
Current or smoked in the last 10 years	1,327	306,081	0.97 (0.86, 1.08)	0.99 (0.88, 1.12)	0.88 (0.72, 1.08)	0.94 (0.76, 1.15)	1.01 (0.85, 1.21)	1.03 (0.86, 1.24)
Adenocarcinomas
Full cohort	847	1,510,027	1.15 (0.95, 1.39)	1.18 (0.97, 1.45)	1.28 (0.89, 1.83)	1.33 (0.92, 1.93)	1.18 (0.87, 1.59)	1.23 (0.89, 1.70)
Never or quit smoking ≥ 10 years	425	1,203,946	1.18 (0.83, 1.67)	1.41 (0.95, 2.09)	1.41 (0.73, 2.72)	1.66 (0.81, 3.42)	1.14 (0.69, 1.86)	1.49 (0.85, 2.63)
^***a***^Models were adjusted for age, time period, and geographic region. ^***b***^Additionally adjusted for BMI, alcohol consumption, physical activity, overall diet quality, smoking status (when not stratified by status) and pack-years, months since quitting smoking, secondhand smoke exposure at home, work, and during childhood, and census-tract median home value and median income.								

Among all lung cancer cases, 44% were adenocarcinoma, 14% were squamous, 14% were small cell, 16% were other histologies (large cell and non-small cell carcinoma, carcinoid, or papillary, mixed sarcoma), and 12% were unknown histology. There were sufficient numbers only of adenocarcinomas to perform subtype-specific analyses. In general, HRs for associations with adenocarcinomas were stronger than corresponding HRs for all lung cancer subtypes combined (Table 2).

In sensitivity analyses, the HRs for PM_2.5_ and all lung cancers, the HRs for associations with adenocarcinomas specifically, and the HRs based on models restricted to never- or long-term former smokers were slightly stronger when analyses were restricted to 66,051 women who did not move residence between 1976 and 1994 (986,370 person-years, 1,441 total cases) (see Supplemental Material, Table S3). Furthermore, our conclusions were unchanged when we used the 24-, 48-, 96-, or 120-month cumulative averages for PM (data not shown).

There were a total of 1,291,229 person-years of follow-up and 1,841 lung cancer cases (1,654 definite) among the 88,596 women with information on distance to road in 1994 (i.e., with a street segment level geocode). Results from basic and multivariable adjusted models of associations with distance to road are presented in Table 3 for each of the different roadway size/distance category combinations. Living within 50 m of an A1 road versus > 200 m from an A1 road was positively associated with lung cancer, although estimates were very unstable due to the small number of cases (*n* = 10). When distance to road was modeled continuously, there was no apparent association with decreasing distance. In sensitivity analyses restricted to women who did not move between 1976 and 1994, results were similar (see Supplemental Material, Table S4).

**Table 3:** HRs (95% CIs) for incident lung cancer 1994–2010 in association with residential proximity to roads in 1994 among 88,596 members of the Nurses’ Health Study.

Exposure category	Cases	Basic^*a*^	Adjusted^*b*^
Full cohort
Distance to A1 (m)
≥ 200	1,799	1.00 (Referent)	1.00 (Referent)
50–199	32	0.70 (0.49, 0.99)	0.73 (0.51, 1.04)
0–49	10	2.38 (1.27, 4.44)	2.01 (1.06, 3.80)
Continuous (per 100 m)	1,841	1.01 (0.95, 1.08)	1.01 (0.95, 1.07)
Distance to A1–A2 (m)
≥ 200	1,699	1.00 (Referent)	1.00 (Referent)
50–199	105	0.93 (0.76, 1.13)	0.93 (0.76, 1.14)
0–49	37	1.14 (0.82, 1.58)	1.02 (0.73, 1.42)
Continuous (per 100 m)	1,841	1.00 (0.96, 1.04)	1.01 (0.97, 1.04)
Distance to A1–A3 (m)
≥ 200	971	1.00 (Referent)	1.00 (Referent)
50–199	558	1.07 (0.96, 1.19)	1.05 (0.94, 1.17)
0–49	312	1.10 (0.97, 1.25)	1.05 (0.92, 1.19)
Continuous (per 100 m)	1,841	0.98 (0.95, 1.00)	0.99 (0.96, 1.02)
Current smoker or smoked in the last ^*10 years*^
Distance to A1 (m)
≥ 200	1,212	1.00 (Referent)	1.00 (Referent)
50–199	19	0.79 (0.48, 1.31)	0.86 (0.52, 1.42)
0–49	6	2.74 (1.19, 6.32)	2.48 (1.04, 5.90)
Continuous (per 100 m)	1,237	1.00 (0.91, 1.09)	0.98 (0.89, 1.07)
Distance to A1–A2 (m)
≥ 200	1,142	1.00 (Referent)	1.00 (Referent)
50–199	69	0.96 (0.73, 1.28)	0.99 (0.75, 1.33)
0–49	26	1.01 (0.62, 1.62)	0.95 (0.58, 1.15)
Continuous (per 100 m)	1,237	1.02 (0.96, 1.08)	1.02 (0.96, 1.08)
Distance to A1–A3 (m)
≥ 200	647	1.00 (Referent)	1.00 (Referent)
50–199	370	1.10 (0.94, 1.28)	1.10 (0.94, 1.29)
0–49	220	1.10 (0.91, 1.33)	1.11 (0.92, 1.34)
Continuous (per 100 m)	1,237	0.98 (0.94, 1.02)	0.97 (0.93, 1.01)
Never-smoker or quit smoking ≥ 10 years
Distance to A1 (m)
≥ 200	587	1.00 (Referent)	1.00 (Referent)
50–199	13	0.86 (0.49, 1.49)	0.90 (0.52, 1.57)
0–49	4	3.15 (1.16, 8.50)	3.26 (1.17, 9.11)
Continuous (per 100 m)	604	0.98 (0.88, 1.09)	0.98 (0.88, 1.09)
Distance to A1–A2 (m)
≥ 200	557	1.00 (Referent)	1.00 (Referent)
50–199	36	0.99 (0.71, 1.39)	1.03 (0.73, 1.45)
0–49	11	1.05 (0.58, 1.91)	1.07 (0.59, 1.96)
Continuous (per 100 m)	604	0.98 (0.91, 1.04)	0.97 (0.90, 1.03)
Distance to A1–A3 (m)
≥ 200	324	1.00 (Referent)	1.00 (Referent)
50–199	188	1.10 (0.91, 1.31)	1.09 (0.91, 1.31)
0–49	92	1.00 (0.79, 1.26)	1.03 (0.81, 1.30)
Continuous (per 100 m)	604	1.00 (0.95, 1.04)	0.99 (0.95, 1.04)
^***a***^Models were adjusted for age, time period, and geographic region. ^***b***^Additionally adjusted for BMI, alcohol consumption, physical activity, overall diet quality, smoking status (when not stratified by status) and pack-years, months since quitting smoking, secondhand smoke exposure at home, work, and during childhood, and census-tract median home value and median income.			

## Discussion

In this large prospective cohort study of women living in the contiguous United States, we observed positive associations between long-term exposures to ambient air pollution (PM_10_, PM_2.5_, and PM_2.5–10_) and incident lung cancer after adjusting for time-varying information on known risk factors, including lifetime smoking history, diet, and physical activity. In the full cohort, a 10-μg/m^3^ increase in the 72-month cumulative average of all three size fractions was associated with modest increases in risk [PM_10_: HR = 1.04 (95% CI: 0.95, 1.14); PM_2.5_: HR = 1.06 (95% CI: 0.91, 1.25); PM_2.5–10_: HR = 1.05 (95% CI: 0.92, 1.20)]. However, associations were stronger when the cohort was restricted to never-smokers and former smokers who had quit at least 10 years before diagnosis, particularly for PM_2.5_ [PM_10_: HR = 1.15 (95% CI: 1.00, 1.32); PM_2.5_: HR = 1.37 (95% CI: 1.06, 1.77); PM_2.5–10_: HR = 1.11 (95% CI: 0.90, 1.37)]. In addition, associations were stronger when only the most common lung cancer subtype, adenocarcinoma, was considered. We also assessed distance to major roadways as a proxy for overall traffic exposures. Living within 50 m of an A1 road (versus > 200 m from an A1 road) was positively associated with lung cancer risk, but numbers of exposed participants were small and there was no evidence of an association with distance from a road modeled as a continuous variable.

Our estimated HRs for a 10-μg/m^3^ increase in PM_2.5_ and PM_10_ in the full cohort are at the lower end of the distribution of associations observed in most of the previous equivalent studies (Beelen et al. 2008a, 2008b; Beeson et al. 1998; Cao and Gao 2012; Carey et al. 2013; Cesaroni et al. 2013; Hales et al. 2013; Hart et al. 2011; Heinrich et al. 2013; Hystad et al. 2013; Jerrett et al. 2013; Katanoda et al. 2011; Krewski et al. 2009; Lepeule et al. 2012; Lipsett et al. 2011; McDonnell et al. 2000; Naess et al. 2007; Nafstad et al. 2003; Nyberg et al. 2000; Pope et al. 2002; Raaschou-Nielsen et al. 2010, 2011, 2013; Turner et al. 2011). These studies have reported associations in ranges of 0.95–1.40 for PM_2.5_ and 0.93–2.40 for PM_10_ when estimated for increments of 10 μg/m^3^. To our knowledge, to date, only one other study has presented assessment of PM_2.5–10_ (Raaschou-Nielsen et al. 2013), and the HR was 1.19 (95% CI: 0.77, 1.82) if expressed as a 10-μg/m^3^ increase.

In its recent assessment of the carcinogenicity of outdoor air pollution in general and particulate matter in particular, IARC determined that the evidence was remarkably consistent in epidemiological studies from Europe, North America, and Asia, in studies of experimental animals, and across a wide range of mechanisms related to cancer (Loomis et al. 2013). IARC determined that for lung cancer the most informative epidemiologic studies were the European Study of Cohorts for Air Pollution Effects (ESCAPE) and the American Cancer Society Study (ACS). ESCAPE combined data from 17 cohort studies based in nine European countries, including 312,944 individuals and 2,095 incident lung cancer cases over 12.8 years of follow-up (Raaschou-Nielsen et al. 2013). Using land-use regression models incorporating data from 2008–2011, they predicted PM exposures at the participants’ baseline address (in the 1990s for most cohorts). They observed associations of lung cancer with PM_10_ (HR = 1.22; 95% CI 1.03, 1.45 per 10 μg/m^3^), PM_2.5_ (HR = 1.18; 95% CI: 0.96, 1.46 per 5 μg/m^3^), and PM_2.5–10_ (HR = 1.09; 95% CI: 0.88, 1.33 per 5 μg/m^3^) after adjusting for sex, smoking variables, secondhand smoke, occupational variables, fruit intake, and area-level SES (Raaschou-Nielsen et al. 2013). The most recent updates of the full ACS Cancer Prevention Study II (CPS-II) study included about 500,000 individuals residing in metropolitan statistical areas (MSAs) throughout the United States with information on air pollution (Krewski et al. 2009; Pope et al. 2002). The investigators estimated MSA-level average baseline exposures to PM_2.5_ from 1979–1983 and toward the end of the follow-up in 1999–2000. HRs were adjusted for sex, age, race, and baseline information on smoking, education, marital status, BMI, alcohol consumption, occupational exposure, and diet. With follow-up from 1982–1998, the adjusted HRs for a 10-μg/m^3^ increase in PM_2.5_ using the baseline average, the 1999–2000 average, or the average of the two time periods were 1.08 (95% CI: 1.01, 1.16), 1.13 (95% CI: 1.04, 1.22), and 1.14 (95% CI: 1.04, 1.23), respectively (Pope et al. 2002). The adjusted HR per 28.8 μg/m^3^ for exposure to PM_10_ averaged from 1987–1996 was not elevated (HR = 0.94; 95% CI: 0.86, 1.02) (Krewski et al. 2009). An additional 2 years of follow-up and extensive consideration of additional individual-level and ecological-level covariates, as well as assessment of autocorrelation, did not materially change these results (Krewski et al. 2009).

There is no clear evidence in the literature of sex differences in the relation of PM with lung cancer. To date, only two other studies have focused specifically on women. In the California Teachers Study, a prospective cohort of 133,479 female public school professionals (20–80 years of age at baseline) residential-level cumulative exposures to PM_2.5_ and PM_10_ were quantified. From 1997 through 2005, 234 and 275 participants with PM_2.5_ and PM_10_ exposure information, respectively, died from lung cancer. Adjusted analyses showed no association with a 10-μg/m^3^ change in PM_2.5_ (HR = 0.95; 95% CI: 0.70–1.28) or PM_10_ (HR = 0.93; 95% CI: 0.81–1.07) (Lipsett et al. 2011). In a German cohort study of 4,800 women, air pollution exposure was assessed for up to 18 years using air monitoring–station data to calculate yearly averages of PM_10_. Adjusted analysis showed an increase of 7 μg/m^3^ PM_10_ was associated with an increased HR for lung cancer mortality (HR = 1.84; 95% CI: 1.23, 2.74) (Heinrich et al. 2013). In other studies that presented results stratified by sex, no clear patterns of effect modification emerged (Abbey et al. 1999; Cesaroni et al. 2013; Katanoda et al. 2011; Naess et al. 2007; Pope et al. 2002).

Our findings of stronger associations when we restricted to never-smokers and participants who had quit at least 10 years before diagnosis are consistent with the majority of the literature. After 26 years of follow-up in the ACS study, the adjusted HR for lung cancer mortality risk among never smokers was 1.27 (95% CI: 1.03, 1.56) for a 10-μg/m^3^ change in PM_2.5_ (1999–2000) (Turner et al. 2011). In ESCAPE, the HRs for a 10-μg/m^3^ change in PM_10_ increased to 1.39 (95% CI: 0.94, 2.06) and for a 5-μg/m^3^ increase in PM_2.5_ to 1.21 (95% CI: 0.61, 2.40) (Raaschou-Nielsen et al. 2013). Although no association between PM and lung cancer mortality was observed in the California Teachers Study, when the population was restricted to never-smokers the adjusted HR for a 10-μg/m^3^ change in PM_2.5_, but not PM_10_, showed an increased risk (PM_2.5_ HR = 1.62; 95% CI 0.83, 3.16; PM_10_ HR = 1.00; 95% CI: 0.75, 1.31) (Lipsett et al. 2011). In the Harvard Six Cities Study (*n* = 8,096), adjusted HRs increased from 1.37 (95% CI: 1.07, 1.75) for a 10-μg/m^3^ change overall, to 1.96 (95% CI: 1.29, 2.99) among former smokers. However, HRs for never- and current smokers were 1.25 (95% CI: 0.54, 2.89) and 1.25 (0.95, 1.64), respectively (Lepeule et al. 2012). Similarly, in a case–control study in Canada, adjusted HRs for the whole study were 1.29 (95% CI: 0.95, 1.76) per 10-μg/m^3^ increase in PM_2.5_, but HRs among never-, former, and current smokers were 0.95 (95% CI: 0.38, 2.34), 1.45 (95% CI: 0.96, 2.19), and 1.17 (95% CI: 0.75, 1.84), respectively (Hystad et al. 2013). In a Japanese cohort of 63,520 participants, all never-smokers were female. The adjusted HR for a 10-μg/m^3^ increase in PM_2.5_ was 1.16 (95% CI: 1.02, 1.33) in the never-smokers, whereas the HR for all females was 1.17 (95% CI: 0.98, 1.39) (Katanoda et al. 2011). Except for the ACS study (1,100 cases), in each individual study the numbers of lung cancer cases who had never smoked was quite small (< 200). Overall, however, because they reduce the potential for residual confounding by smoking, these analyses among never- and former smokers increase our confidence that exposure to air pollution is independently associated with lung cancer.

Very few studies have estimated associations of air pollution with histological subtypes of lung cancer. Hystad et al. (2013) reported increased risk of adenocarcinoma for each 10-μg/m^3^ increase in PM_2.5_ [odds ratio (OR) = 1.27; 95% CI: 0.84, 1.90]. Similar to our study, in ESCAPE results were stronger when the case definition was restricted to adenocarcinomas [5-μg/m^3^ increase in PM_2.5_ HR = 1.55 (95% CI: 1.05, 2.29) and 10-μg/m^3^ increase in PM_10_ exposure HR = 1.51 (95% CI: 1.10, 2.08)] (Raaschou-Nielsen et al. 2013). In a hospital-based case–control study of > 1,200 patients in northern Spain, with residential area as a proxy for air pollution levels, residents in urban areas showed significantly increased risks for adenocarcinoma (OR = 1.92; 95% CI: 1.09, 3.38) compared with those in rural areas (López-Cima et al. 2011). Adenocarcinomas are the lung cancer subtype most commonly observed among nonsmokers (Schuller 2002), and time-trend and geographic studies have also suggested associations of this subtype with ambient air pollution (Chen et al. 2007, 2009).

Conclusions from studies specifically assessing the association of lung cancer with traffic exposures have been inconclusive. Six previous studies in Europe and Canada have looked at measures of distance to roadway or traffic intensity (Beelen et al. 2008a, 2008b; Cesaroni et al. 2013; Hystad et al. 2013; Raaschou-Nielsen et al. 2011, 2013; Vineis et al. 2006). Similar to our findings, the results have suggested a modest association, though the different metrics are difficult to compare and no results are statistically significant. Four studies, all in Europe, have modeled NO_2_ or NO_x_ specifically from traffic sources (Nafstad et al. 2003; Nyberg et al. 2000; Raaschou-Nielsen et al. 2010, 2011). Overall, these studies indicate a possible contribution from traffic, but again they are difficult to compare due to differences in exposure assessment.

Our study has a number of limitations. We used a spatiotemporal model to assign monthly residential-level PM exposures for each participant. However, we do not account for differences in time–activity patterns, time spent outdoors, or time spent at the residence. Additionally, because of a paucity of monitoring for PM_2.5_ before 1999, our models for PM_2.5_ and PM_2.5–10_ in the earlier years are less precise than our models in the later years. Although we had detailed residential history information during the course of the study when the women were all at least 58 years old, we were unable to assess exposures throughout the life course. Another source of exposure measurement error is the temporal mismatch of our road layer with the address information used in the distance to road analyses, which may partially explain our generally null results. Even though our study was conducted within a large cohort of women with a large number of total cases, we were limited by the small number of cases among certain subgroups of particular interest, including never-smokers, people living close to roadways, or cases of histological subtypes other than adenocarcinomas. As with all studies, residual confounding, particularly by active and passive smoking, is of concern. We controlled for secondhand smoke exposures; however, this information was available only at one time point and was not available for a large number of participants. Although modification by educational attainment has been reported (Raaschou-Nielsen et al. 2011), this cohort of predominantly Caucasian nurses allows very limited exploration of the influence of SES or race, although controlling for individual- and area-level SES along with other risk factors made little difference to the results.

This study also has several strengths, including our ability to control for time-varying exposures after baseline. Additionally, we were able to examine the effects of adjusting for a number of different parameterizations of smoking, incorporating time since quitting as well as duration and intensity. We were also able to adjust for exposures to secondhand smoke. Although the survival rate of lung cancer is low, we assessed lung cancer incidence as opposed to mortality. Finally, we had sufficient adenocarcinomas to be able to specifically examine this subtype of interest.

## Conclusions

In the largest study of incident lung cancers among women to date, positive associations were observed with average PM exposures in the previous 72 months. Associations were stronger when analyses were restricted to adenocarcinomas, or to never- and long-term former smokers. This study provides additional support of an association of air pollution exposure and lung cancer, particularly among nonsmokers.

## Supplemental Material

(1.4 MB) PDF

## References

[r1] Abbey DE, Nishino N, McDonnell WF, Burchette RJ, Knutsen SF, Beeson WL (1999). Long-term inhalable particles and other air pollutants related to mortality in nonsmokers.. Am J Respir Crit Care Med.

[r2] Adar SD, Kaufman JD (2007). Cardiovascular disease and air pollutants: evaluating and improving epidemiological data implicating traffic exposure.. Inhal Toxicol.

[r3] Akaike H (1974). A new look at the statistical model identification.. IEEE Trans Automat Contr.

[r4] Beelen R, Hoek G, van den Brandt PA, Goldbohm RA, Fischer P, Schouten LJ (2008a). Long-term exposure to traffic-related air pollution and lung cancer risk.. Epidemiology.

[r5] Beelen R Hoek G van den Brandt PA Goldbohm RA Fischer P Schouten LJ 2008b Long-term effects of traffic-related air pollution on mortality in a Dutch cohort (NLCS-AIR study). Environ Health Perspect 116 196 202; 10.1289/ehp.10767 18288318 PMC2235230

[r6] Beeson WL, Abbey DE, Knutsen SF (1998). Long-term concentrations of ambient air pollutants and incident lung cancer in California adults: results from the AHSMOG study.. Environ Health Perspect.

[r7] Cao Y, Gao H (2012). Prevalence and causes of air pollution and lung cancer in Xuanwei City and Fuyuan County, Yunnan Province, China.. Front Med.

[r8] Carey IM, Atkinson RW, Kent AJ, van Staa T, Cook DG, Anderson HR (2013). Mortality associations with long-term exposure to outdoor air pollution in a national English cohort.. Am J Respir Crit Care Med.

[r9] Cesaroni G Badaloni C Gariazzo C Stafoggia M Sozzi R Davoli M 2013 Long-term exposure to urban air pollution and mortality in a cohort of more than a million adults in Rome. Environ Health Perspect 121 324 331; 10.1289/ehp.1205862 23308401 PMC3621202

[r10] Chen F, Cole P, Bina WF (2007). Time trend and geographic patterns of lung adenocarcinoma in the United States, 1973–2002.. Cancer Epidemiol Biomarkers Prev.

[r11] Chen F, Jackson H, Bina WF (2009). Lung adenocarcinoma incidence rates and their relation to motor vehicle density.. Cancer Epidemiol Biomarkers Prev.

[r12] Chiuve SE, Fung TT, Rimm EB, Hu FB, McCullough ML, Wang M (2012). Alternative dietary indices both strongly predict risk of chronic disease.. J Nutr.

[r13] Dockery DW, Pope CA, Xu X, Spengler JD, Ware JH, Fay ME (1993). An association between air pollution and mortality in six U.S. Cities.. N Engl J Med.

[r14] Filleul L, Rondeau V, Vandentorren S, Le Moual N, Cantagrel A, Annesi-Maesano I (2005). Twenty five year mortality and air pollution: results from the French PAARC survey.. Occup Environ Med.

[r15] Hales S, Blakely T, Woodward A (2013). Air pollution and mortality in New Zealand: cohort study.. J Epidemiol Community Health.

[r16] Hart JE, Garshick E, Dockery DW, Smith TJ, Ryan L, Laden F (2011). Long-term ambient multipollutant exposures and mortality.. Am J Respir Crit Care Med.

[r17] Heinrich J, Thiering E, Rzehak P, Krämer U, Hochadel M, Rauchfuss KM (2013). Long-term exposure to NO_2_ and PM_10_ and all-cause and cause-specific mortality in a prospective cohort of women.. Occup Environ Med.

[r18] Hystad P, Demers PA, Johnson KC, Carpiano RM, Brauer M (2013). Long-term residential exposure to air pollution and lung cancer risk.. Epidemiology.

[r19] Jerrett M, Burnett RT, Beckerman BS, Turner MC, Krewski D, Thurston G (2013). Spatial analysis of air pollution and mortality in California.. Am J Respir Crit Care Med.

[r20] Karner AA, Eisinger DS, Niemeier DA (2010). Near-roadway air quality: synthesizing the findings from real-world data.. Environ Sci Technol.

[r21] Katanoda K, Sobue T, Satoh H, Tajima K, Suzuki T, Nakatsuka H (2011). An association between long-term exposure to ambient air pollution and mortality from lung cancer and respiratory diseases in Japan.. J Epidemiol.

[r22] Krewski D, Jerrett M, Burnett RT, Ma R, Hughes E, Shi Y (2009). Extended follow-up and spatial analysis of the American Cancer Society study linking particulate air pollution and mortality.. Res Rep Health Eff Inst.

[r23] Lepeule J Laden F Dockery D Schwartz J 2012 Chronic exposure to fine particles and mortality: an extended follow-up of the Harvard Six Cities study from 1974 to 2009. Environ Health Perspect 120 965 970; 10.1289/ehp.1104660 22456598 PMC3404667

[r24] Lipfert FW, Baty JD, Miller JP, Wyzga RE (2006). PM_2.5_ constituents and related air quality variables as predictors of survival in a cohort of U.S. military veterans.. Inhal Toxicol.

[r25] Lipfert FW, Wyzga RE (2008). On exposure and response relationships for health effects associated with exposure to vehicular traffic.. J Expo Sci Environ Epidemiol.

[r26] Lipfert FW, Wyzga RE, Baty JD, Miller JP (2008). Vehicular traffic effects on survival within the Washington University-EPRI veterans cohort: new estimates and sensitivity studies.. Inhal Toxicol.

[r27] Lipsett MJ, Ostro BD, Reynolds P, Goldberg D, Hertz A, Jerrett M (2011). Long-term exposure to air pollution and cardiorespiratory disease in the California Teachers Study cohort.. Am J Respir Crit Care Med.

[r28] Loomis D, Grosse Y, Lauby-Secretan B, El Ghissassi F, Bouvard V, Benbrahim-Tallaa L (2013). The carcinogenicity of outdoor air pollution.. Lancet Oncol.

[r29] López-Cima MF García-Pérez J Pérez-Gómez B Aragonés N López-Abente G Tardón A 2011 Lung cancer risk and pollution in an industrial region of northern Spain: a hospital-based case-control study. Int J Health Geogr 10 10; 10.1186/1476-072X-10-10 21266041 PMC3040690

[r30] McDonnell WF, Nishino-Ishikawa N, Petersen FF, Chen LH, Abbey DE (2000). Relationships of mortality with the fine and coarse fractions of long-term ambient PM_10_ concentrations in nonsmokers.. J Expo Anal Environ Epidemiol.

[r31] Naess Ø, Nafstad P, Aamodt G, Claussen B, Rosland P (2007). Relation between concentration of air pollution and cause-specific mortality: four-year exposures to nitrogen dioxide and particulate matter pollutants in 470 neighborhoods in Oslo, Norway.. Am J Epidemiol.

[r32] Nafstad P, Håheim LL, Oftedal B, Gram F, Holme I, Hjermann I (2003). Lung cancer and air pollution: a 27 year follow up of 16 209 Norwegian men.. Thorax.

[r33] Nyberg F, Gustavsson P, Järup L, Bellander T, Berglind N, Jakobsson R (2000). Urban air pollution and lung cancer in Stockholm.. Epidemiology.

[r34] Pope CA, Burnett RT, Thun MJ, Calle EE, Krewski D, Ito K (2002). Lung cancer, cardiopulmonary mortality, and long-term exposure to fine particulate air pollution.. JAMA.

[r35] Raaschou-Nielsen O, Andersen ZJ, Beelen R, Samoli E, Stafoggia M, Weinmayr G (2013). Air pollution and lung cancer incidence in 17 European cohorts: Prospective analyses from the European Study of Cohorts for Air Pollution Effects (ESCAPE).. Lancet Oncol.

[r36] Raaschou-Nielsen O Andersen ZJ Hvidberg M Jensen SS Ketzel M Sørensen M 2011 Lung cancer incidence and long-term exposure to air pollution from traffic. Environ Health Perspect 119 860 865; 10.1289/ehp.1002353 21227886 PMC3114823

[r37] Raaschou-Nielsen O, Bak H, Sørensen M, Jensen SS, Ketzel M, Hvidberg M (2010). Air pollution from traffic and risk for lung cancer in three Danish cohorts.. Cancer Epidemiol Biomarkers Prev.

[r38] Sahlodin AM, Sotudeh-Gharebagh R, Zhu Y (2007). Modeling of dispersion near roadways based on the vehicle-induced turbulence concept.. Atmos Environ.

[r39] Schuller HM (2002). Mechanisms of smoking-related lung and pancreatic adenocarcinoma development.. Nat Rev Cancer.

[r40] Spengler JD, Koutrakis P, Dockery DW, Raizenne M, Speizer FE (1996). Health effects of acid aerosols on north American children: air pollution exposures.. Environ Health Perspect.

[r41] Suh HH, Nishioka Y, Allen GA, Koutrakis P, Burton RM (1997). The metropolitan acid aerosol characterization study: results from the summer 1994 Washington, D.C. field study.. Environ Health Perspect.

[r42] Turner MC, Krewski D, Pope CA, Chen Y, Gapstur SM, Thun MJ (2011). Long-term ambient fine particulate matter air pollution and lung cancer in a large cohort of never-smokers.. Am J Respir Crit Care Med.

[r43] U.S. Census Bureau. (2001). Census 2000 TIGER/Line® Files: Technical Documentation.. http://www.census.gov/geo/maps-data/data/pdfs/tiger/rd_2ktiger/tgrrd2k.pdf.

[r44] U.S. EPA (U.S. Environmental Protection Agency). (2009). Air Quality System.. http://www.epa.gov/ttn/airs/airsaqs/.

[r45] Villeneuve PJ, Jerrett M, Su J, Burnett RT, Chen H, Brook J (2013). A cohort study of intra-urban variations in volatile organic compounds and mortality, Toronto, Canada.. Environ Pollut.

[r46] Vineis P, Hoek G, Krzyzanowski M, Vigna-Taglianti F, Veglia F, Airoldi L (2006). Air pollution and risk of lung cancer in a prospective study in Europe.. Int J Cancer.

[r47] Visibility Information Exchange Web System. (2009). Home page.. http://vista.cira.colostate.edu/views/.

[r48] Weuve J, Puett RC, Schwartz J, Yanosky JD, Laden F, Grodstein F (2012). Exposure to particulate air pollution and cognitive decline in older women.. Arch Intern Med.

[r49] Yanosky JD Paciorek CJ Laden F Hart J Puett R Suh HH In press Spatio-temporal modeling of particulate air pollution in the conterminous United States using geographic and meteorological predictors. Environ Health. 10.1186/1476-069X-13-63 PMC4137272 25097007

[r50] Yanosky JD, Paciorek CJ, Schwartz J, Laden F, Puett R, Suh HH (2008). Spatio-temporal modeling of chronic PM_10_ exposure for the Nurses’ Health Study.. Atmos Environ.

[r51] Yanosky JD Paciorek CJ Suh HH 2009 Predicting chronic fine and coarse particulate exposures using spatiotemporal models for the northeastern and midwestern United States. Environ Health Perspect 117 522 529; 10.1289/ehp.11692 19440489 PMC2679594

[r52] Zhu Y, Hinds WC, Kim S, Sioutas C (2002). Concentration and size distribution of ultrafine particles near a major highway.. J Air Waste Manag Assoc.

