# Supplementary material for: Particulate Matter Air Pollution Exposure, Distance to Road, and Incident Lung Cancer in the Nurses’ Health Study Cohort
Source: Environ Health Perspect. 2014 Jun 3;122(9):926–32. doi: 10.1289/ehp.1307490 (PMC4154215; doi:10.1289/ehp.1307490)
Supplement: (1.4 MB) PDF [file ehp.1307490.s001.pdf]

Environmental Health Perspectives doi:10.1289/ehp.1307490

Supplemental Material

Particulate Matter Air Pollution Exposure, Distance to Road, and Incident Lung Cancer in the Nurses' Health Study Cohort

Robin C. Puett, Jaime E. Hart, Jeff D. Yanosky, Donna Spiegelman, Molin Wang, Jared A. Fisher, Biling Hong, and Francine Laden

Table of Contents **Page**

Supplemental Methods **2**

Table S1. Distributions of 72-month cumulative average PM exposures (μg/m^3) overall and by region for 103,650 members of the Nurses' Health Study **3**

Table S2. Spearman correlations of the 24-, 48-, and 72-month cumulative average exposures to PM_{10} $PM_{2.5}$ and $PM_{2.5-10}$ **4**

Table S3. Hazard ratios and 95% confidence intervals of the association of incident lung cancer 1994-2010 per 1 μg/m increase in 72-month cumulative Average particulate matter exposures among 66,051 members of the Nurses' Health Study who did not move between 197 and 1994 **5**

Table S4. Hazard Ratios and 95% CIs for incident lung cancer 1994-2010 in association with residential proximity to roads in 1994 among 57,495 members of the Nurses' Health Study who did not move between 197 and 1994 **6**

Figure S1. Residential address locations 1976, 1988-2010 of the 103,650 members of the Nurses' Health Study included in the analyses of PM effects **8**

Supplemental Methods

Some data used in this analysis was provided by the following state cancer registries: Alabama, Arizona, Arkansas, California, Colorado, Connecticut , Delaware, Florida, Georgia, Idaho, Illinois, Indiana, Iowa, Kentucky, Louisiana, Maine, Maryland, Massachusetts, Michigan, Nebraska, New Hampshire, New Jersey, New York, North Carolina, North Dakota, Ohio, Oklahoma, Oregon, Pennsylvania, Rhode Island, South Carolina, Tennessee, Texas, Virginia, Washington, Wyoming.

Table S1. Distributions of 72-month cumulative average PM exposures (μg/m^3) overall and by region for 103,650 members of the Nurses' Health Study.

Exposure	Overall	Northeast	Midwest	West	South
$PM_{2.5}$					
Mean	13.11	13.18	14.29	12.93	11.89
Standard Deviation	3.03	2.58	2.63	4.60	2.56
Median	12.93	12.02	14.52	12.10	11.52
Interquartile Range	4.12	3.62	3.33	6.48	3.48
Minimum	1.51	3.08	2.98	1.15	2.40
Maximum	29.81	25.13	24.27	29.81	23.97
PM_{10}					
Mean	21.58	19.98	22.23	27.35	21.14
Standard Deviation	5.95	4.50	5.09	9.61	3.36
Median	20.68	19.45	22.10	25.81	20.89
Interquartile Range	6.59	6.01	6.83	13.78	4.25
Minimum	3.17	5.91	6.27	3.17	5.03
Maximum	74.79	55.60	55.65	74.79	58.83
$PM_{2.5-10}$					
Mean	8.47	6.79	7.93	14.42	9.24
Standard Deviation	4.20	2.33	3.13	6.18	2.97
Range	55.74	42.76	43.45	55.74	42.29
Interquartile Range	4.53	2.94	4.38	7.29	3.83
Minimum	0.06	0.89	0.73	0.06	42.97
Maximum	55.79	43.65	44.19	55.79	0.67

Table S2. Spearman correlations of the 24-, 48-, and 72-month cumulative average exposures to PM_{10}, $PM_{2.5}$, and $PM_{2.5-10}$.

Exposure	$PM_{2.5}$: 72 month	$PM_{2.5}$: 48 month	$PM_{2.5}$: 24 month	PM_{10}: 72 month	PM_{10}: 48 month	PM_{10}: 24 month	$PM_{2.5-10}$: 72 month	$PM_{2.5-10}$: 48 month	$PM_{2.5-10}$: 24 month
$PM_{2.5}$: 72 month	1	0.99	0.96	0.75	0.74	0.71	0.34	0.32	0.29
$PM_{2.5}$: 48 month		1	0.98	0.73	0.74	0.71	0.33	0.32	0.29
$PM_{2.5}$: 24 month			1	0.71	0.72	0.72	0.31	0.30	0.28
PM_{10}: 72 month				1	0.99	0.97	0.88	0.86	0.83
PM_{10}: 48 month					1	0.98	0.87	0.87	0.85
PM_{10}: 24 month						1	0.86	0.87	0.87
$PM_{2.5-10}$: 72 month							1	0.99	0.97
$PM_{2.5-10}$: 48 month								1	0.98
$PM_{2.5-10}$: 24 month									1

Table S3. Hazard ratios and 95% confidence intervals of the association of incident lung cancer 1994-2010 per 10 μg/m^3 increase in 72-month cumulative Average particulate matter exposures among 66,051 members of the Nurses' Health Study who did not move between 1976 and 1994.

Case Definition/Cohort	Cases	Person-Years	PM_{10}: Basic[a]	PM_{10}: Adjusted[b]	$PM_{2.5}$: Basic[a]	$PM_{2.5}$: Adjusted[b]	$PM_{2.5\text{-}10}$:Basic[a]	$PM_{2.5\text{-}10}$: Adjusted[b]
All Cases								
Full Cohort	1,441	986,370	1.08 (0.95, 1.23)	1.04 (0.91, 1.19)	1.12 (0.89, 1.42)	1.13 (0.89, 1.45)	1.11 (0.90, 1.36)	1.01 (0.82, 1.25)
Never Smokers	115	441,659	1.12 (0.72, 1.72)	1.04 (0.68, 1.60)	1.49 (0.67, 3.30)	1.37 (0.61, 3.05)	0.98 (0.49, 1.96)	0.89 (0.46, 1.74)
Never or Quit Smoking at Least 10 Yrs	553	785,152	1.08 (0.88, 1.33)	1.14 (0.92, 1.40)	1.20 (0.83, 1.74)	1.35 (0.92, 1.97)	1.06 (0.77, 1.46)	1.05 (0.76, 1.45)
Current or Smoked in the Last 10 Yrs	888	201,218	0.99 (0.83, 1.19)	1.02 (0.85, 1.22)	0.95 (0.69, 1.31)	1.05 (0.75, 1.45)	1.02 (0.77, 1.36)	1.03 (0.77, 1.37)
Adenocarcinomas								
Full Cohort	557	986,370	1.65 (1.12, 2.41)	1.86 (1.21, 2.86)	2.93 (1.49, 5.75)	3.51 (1.66, 7.42)	1.39 (0.80, 2.41)	1.58 (0.84, 3.00)
Never or Quit Smoking at Least 10 Yrs	277	785,152	1.17 (0.66, 2.06)	2.00 (0.82, 4.90)	2.04 (0.71, 5.87)	2.71 (0.65, 11.30)	0.91 (0.43, 1.93)	2.16 (0.54, 8.64)

[a]Models adjusted for age, time period, and geographic region. [b]Additionally adjusted for BMI, alcohol consumption, physical activity, overall diet quality, smoking status (when not stratified by status) and pack years , months since quit smoking, second hand smoke exposure at home, work and during childhood, and Census tract median home value and median income

Table S4. Hazard Ratios and 95% CIs for incident lung cancer 1994-2010 in association with residential proximity to roads in 1994 among 57,495 members of the Nurses' Health Study who did not move between 1976 and 1994.

Exposure Category	Cases	Basic[a]	Adjusted[b]
**Full Cohort**			
Distance to A1 (m)			
200+	1,230	1.00	1.00
50-199	25	0.87 (0.58, 1.32)	0.96 (0.63, 1.47)
0-49	5	2.22 (0.91, 5.41)	2.34 (0.93, 5.87)
Continuous (per 100 m)	1,260	1.00 (0.92, 1.09)	0.98 (0.90, 1.06)
Distance to A1-A2 (m)			
200+	1,151	1.00	1.00
50-199	85	1.13 (0.89, 1.44)	1.17 (0.92, 1.49)
0-49	24	1.15 (0.73, 1.81)	1.05 (0.66, 1.68)
Continuous (per 100 m)	1,260	0.98 (0.93, 1.03)	0.98 (0.93, 1.03)
Distance to A1-A3 (m)			
200+	626	1.00	1.00
50-199	415	1.11 (0.97, 1.28)	1.12 (0.97, 1.30)
0-49	219	1.10 (0.92, 1.31)	1.09 (0.91, 1.31)
Continuous (per 100 m)	1,260	0.98 (0.94, 1.01)	0.80 (0.94, 1.02)
**Current or Smoked in the Last 10 Yrs**			
Distance to A1 (m)	836	1.00	1.00
200+	15	0.94 (0.52, 1.68)	0.97 (0.54, 1.75)
50-199	3	3.10 (0.93, 10.30)	2.38 (0.64, 8.77)
0-49	854	0.97 (0.77, 1.27)	0.97 (0.86, 1.08)
Continuous (per 100 m)			
Distance to A1-A2 (m)	781	1.00	1.00
200+	55	1.20 (0.87, 1.66)	1.17 (0.84, 1.63)
50-199	18	1.18 (0.67, 2.07)	1.13 (0.63, 2.03)
0-49	854	0.99 (0.92, 1.06)	1.00 (0.93, 1.07)
Continuous (per 100 m)			
Distance to A1-A3 (m)	426	1.00	1.00
200+	271	1.09 (0.90, 1.32)	1.07 (0.88, 1.30)
50-199	157	1.13 (0.90, 1.42)	1.12 (0.89, 1.42)
0-49	854	0.97 (0.92, 1.02)	0.97 (0.92, 1.02)
Continuous (per 100 m)			
**Never or Quit Smoking at Least 10 Yrs**			
Distance to A1 (m)	394	1.00	1.00
200+	10	0.90 (0.48, 1.69)	0.97 (0.52, 1.83)
50-199	2	2.30 (0.56, 9.35)	2.29 (0.53, 9.97)
0-49	406	1.01 (0.88, 1.14)	1.00 (0.88, 1.14)
Continuous (per 100 m)			

Exposure Category	Cases	Basic[a]	Adjusted[b]
Distance to A1-A2 (m)	370	1.00	1.00
200+	30	1.12 (0.77, 1.63)	1.19 (0.81, 1.73)
50-199	6	0.86 (0.38, 1.92)	0.92 (0.40, 2.09)
0-49	406	0.97 (0.89, 1.05)	0.96 (0.88, 1.04)
Continuous (per 100 m)			
Distance to A1-A3 (m)	200	1.00	1.00
200+	144	1.17 (0.94, 1.45)	1.17 (0.94, 1.46)
50-199	62	0.98 (0.73, 1.30)	1.02 (0.77, 1.37)
0-49	406	1.00 (0.95, 1.06)	0.99 (0.94, 1.05)
Continuous (per 100 m)			

[a]Models adjusted for age, time period, and geographic region. [b]Additionally adjusted for BMI, alcohol consumption, physical activity, overall diet quality, smoking status (when not stratified by status) and pack years, months since quit smoking, second hand smoke exposure at home, work, and during childhood, and Census tract median home value and median income.

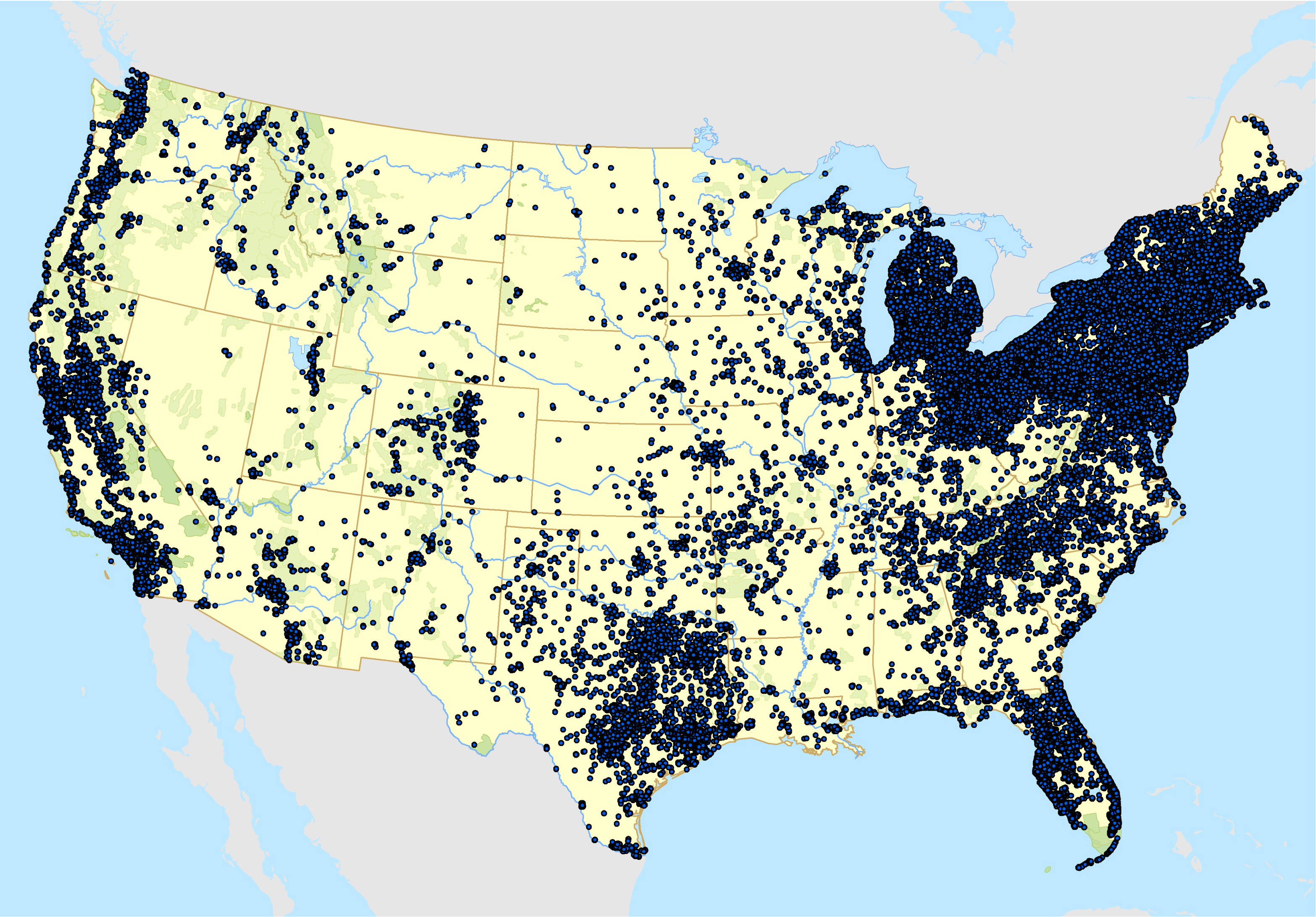

Figure S1. Residential address locations 1976, 1988-2010 of the 103,650 members of the Nurses' Health Study included in the analyses of PM effects.
